# Agricultural use of Burkholderia (Pseudomonas) cepacia: a threat to human health?

**DOI:** 10.3201/eid0402.980209

**Published:** 1998

**Authors:** A Holmes, J Govan, R Goldstein

**Affiliations:** Imperial College School of Medicine, Hammersmith Hospital, London, United Kingdom. aholmes@rpms.ac.uk

## Abstract

In the past 2 decades, Burkholderia cepacia has emerged as a human pathogen causing numerous outbreaks, particularly among cystic fibrosis (CF) patients. One highly transmissible strain has spread across North America and Britain, and another between hospitalized CF and non-CF patients. Meanwhile, the organism has been developed as a biopesticide for protecting crops against fungal diseases and has potential as a bioremediation agent for breaking down recalcitrant herbicides and pesticides. However, B. cepacia is inherently resistant to multiple antibiotics; selection of strains "safe" for environmental application is not at present possible phenotypically or genotypically; molecular epidemiology and phylogenetic studies demonstrate that highly transmissible strains emerge randomly; and the organism has a capacity for rapid mutation and adaptation (facilitated by numerous insertion sequences), and a large, complex genome divided into separate chromosomes. Therefore, the widespread agricultural use of B. cepacia should be approached with caution.


### Agricultural Use of *Burkholderia (Pseudomonas) cepacia*: A Threat to Human Health?

**Alison Holmes,* John Govan,† and Richard Goldstein‡**
*Imperial College School of Medicine, Hammersmith Hospital, London, United Kingdom; †University of Edinburgh Medical School, Edinburgh, United Kingdom; and ‡Maxwell Finland Laboratory for Infectious Diseases, BostonUniversity School of Medicine, Boston, Massachusetts, USA

In the past 2 decades, *Burkholderia cepacia* has emerged as a human pathogen causing numerous outbreaks, particularly among cystic fibrosis (CF) patients. One highly transmissible strain has spread across North America and Britain, and another between hospitalized CF and non-CF patients. Meanwhile, the organism has been developed as a biopesticide for protecting crops against fungal diseases and has potential as a bioremediation agent for breaking down recalcitrant herbicides and pesticides. However, *B. cepacia* is inherently resistant to multiple antibiotics; selection of strains "safe" for environmental application is not at present possible phenotypically or genotypically; molecular epidemiology and phylogenetic studies demonstrate that highly transmissible strains emerge randomly; and the organism has a capacity for rapid mutation and adaptation (facilitated by numerous insertion sequences), and a large, complex genome divided into separate chromosomes. Therefore, the widespread agricultural use of *B. cepacia* should be approached with caution.

*Burkholderia* (previously known as *Pseudomonas) cepacia*, a nutritionally versatile, gram-negative organism, was first described in 1949 by Walter Burkholder of Cornell University, as the phytopathogen responsible for a bacterial rot of onions (1) (Figure 1). Ironically, *B. cepacia* is now being considered by agricultural microbiologists as an agent to promote crop growth.

*B. cepacia* is inherently resistant to multiple antibiotics, can metabolize diverse substrates, and is found in soil and in moist environments. The organism has a particular predilection for the lung in patients with cystic fibrosis (CF) and has emerged as an important opportunistic human pathogen in hospitalized and immunocompromised patients (2,3).

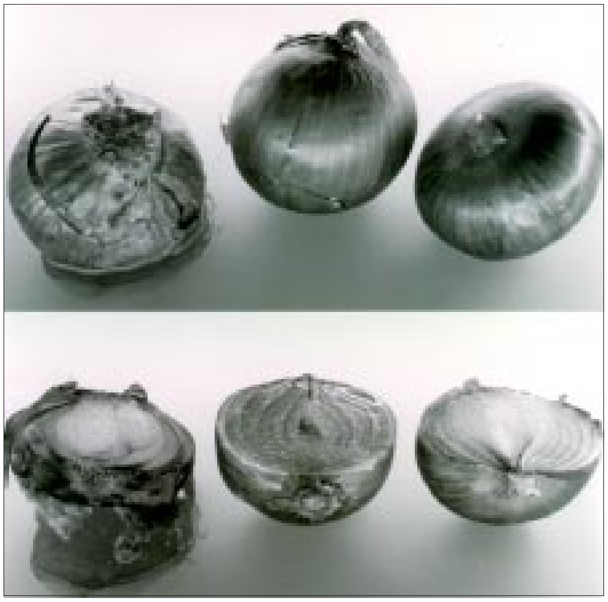

Figure 1. *B. cepacia* causes an onion rot known as slippery skin (1). The onions shown were inoculated with three strains of *B. cepacia*. Rot occurred in onion1 (left), which was inoculated with strain originally isolated from onions. Rot did not occur with environmental isolates tested or with strains from CF lung.

Address for correspondence: Alison Holmes, Department of Infectious Diseases, Hammersmith Hospital, Du Cane Rd, London W12 0NN, United Kingdom; fax: 44-181-383-3394; e-mail: aholmes@rpms.ac.uk.

### *B. cepacia* as a Human Pathogen

### In CF Patients

CF is a recessively transmitted genetic disease that occurs in approximately 1 in 2,500 Caucasians (carrier frequency of 1 in 25). The condition is characterized by a generalized dysfunction of the exocrine glands, giving rise to a broad spectrum of clinical syndromes, especially malabsorption due to pancreatic insufficiency and chronic progressive lung disease giving rise to bronchiectasis. *B. cepacia* is associated with increased illness and death among CF patients. In the early 1980s, the organism emerged as a major threat, causing superinfection in as many as 40% of patients in some CF centers (4-6). While in some patients indolent pulmonary infection occurs with only gradual deterioration in lung function similar to that associated with *Pseudomonas aeruginosa*, approximately 35% of *B. cepacia*–infected patients contract accelerated pulmonary deterioration or fulminant, necrotizing pneumonia with rapidly fatal bacteremia (3,7-10), sometimes referred to as "*cepacia* syndrome" (7). Unlike infection with *P. aeruginosa*, *B. cepacia* infection significantly increases death rates among CF patients (11) at all levels of lung function.

Over the last 20 years, CF survival rates have increased as a result of improved treatment, the median length of survival increasing from early childhood to more than 29 years. As a result, approximately one third of CF patients are adults. Pulmonary infection causes the most illness and ultimately more than 90% of CF-related deaths. *B. cepacia*'s emergence as a pathogen coincided with social and medical grouping of CF patients in specialized units, clinics, and social groups. Studies subsequently demonstrated that social contact between CF patients was an important factor in transmission of *B. cepacia* (12).

The threat of *B. cepacia* infection led to severe control measures, affecting not only the treatment but also the social and family lives of CF patients. CF centers adopted stringent infection control policies, assuming that all *B. cepacia* isolates were highly transmissible (13,14). CF summer camps in North America were closed, and many lung transplant centers ceased to accept *B. cepacia*–infected CF patients as transplant candidates. Newly formed national and international associations for CF adults and CF families (providing conferences, holidays, and support groups) addressed the issue of *B. cepacia* transmission by abandoning many activities and adopting exclusion and segregation measures (13).

Numerous CF-associated *B. cepacia* epidemics have now been described, and the epidemic strains have been characterized (15-18). One particular highly transmissible strain, epidemically spread within and between CF centers on both sides of the Atlantic, carries the *cblA* gene (18). This gene encodes for the major structural subunit of unique mucin binding cable pili (4). These enormously long pili (radiating 2 to 4 microns) are peritrichously arranged and are intertwined to form cablelike structures that adhere to CF mucin (4,18) (Figure 2). This *cblA*+ strain has spread across Canada and has now been isolated in 50% of CF centers in the United Kingdom (19). Another strain of *B. cepacia* has spread among CF centers in four regions of France (20).

However, it has become clear that transmissibility varies markedly from strain to strain, and that most strains are not involved in epidemics but appear to be independently acquired and show no evidence of transmission. For example, in 8 years no cases of transmission were detected at the University of North Carolina CF center, despite clinical and social contact between patients and the absence of stringent infection control measures (21). Independent acquisition of *B. cepacia* with no evidence of transmission between CF patients was also reported from Denmark (22). Lack of transmission of some strains has also been observed between siblings with CF (23).

### In Patients Without CF

*B. cepacia* was first reported as a human pathogen causing endocarditis in the 1950s. Since then the organism has caused numerous catheter-associated urinary tract infections, wound infections, and intravenous catheter–associated bacteremias. In 1971, it was reported as the causative organism of "foot rot" in U.S. troops on swamp training exercises in northern Florida; it was also isolated from troops serving in the Mekong Delta, Vietnam (24).

In the 1980s the number of nosocomial infections with *B. cepacia* increased markedly, with deaths particularly associated with lung infections (25). Numerous small focal hospital outbreaks involving non-CF patients have usually been due to a contaminated common source, such as disinfectant preparations or

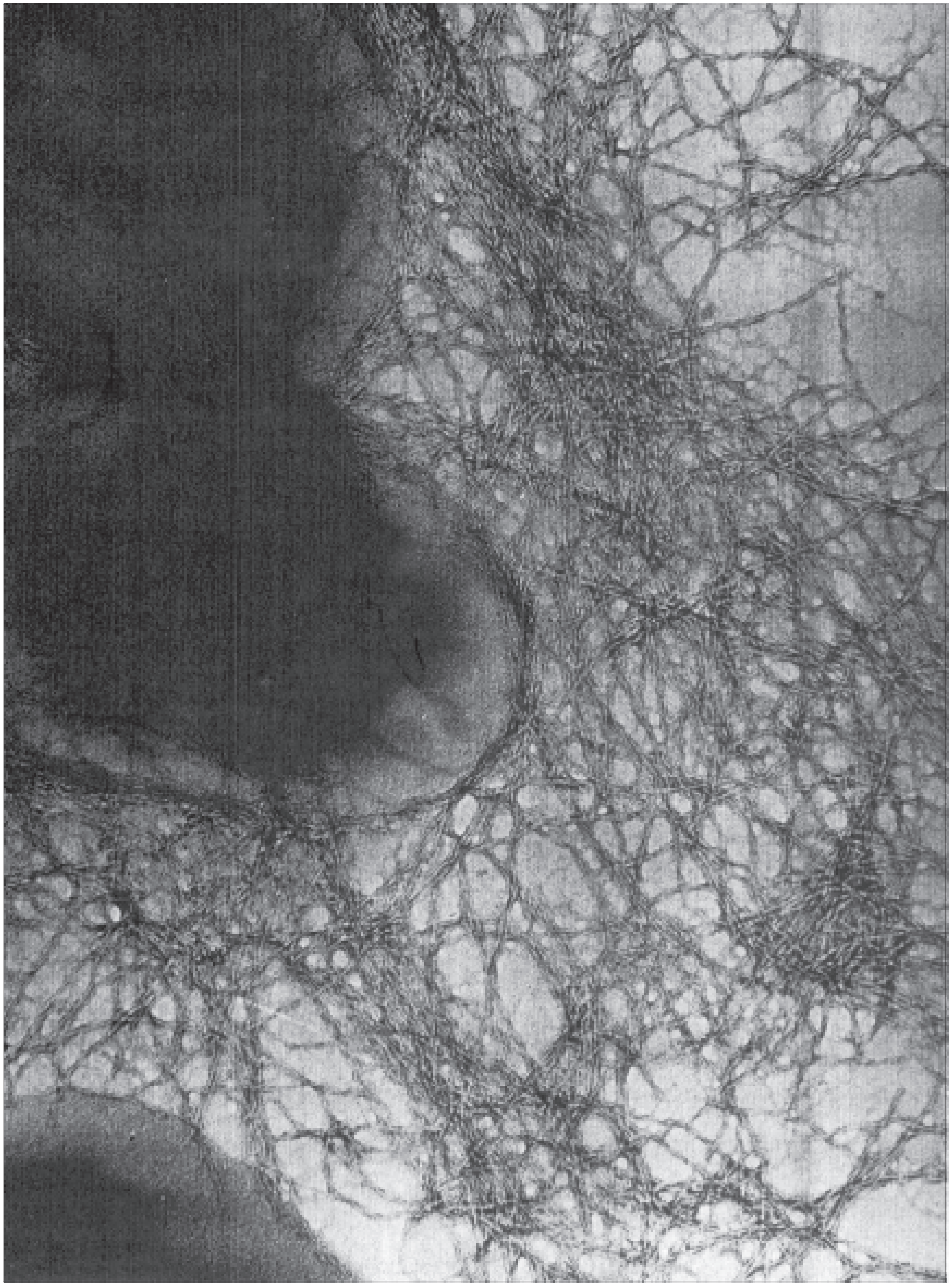

Figure 2. Transmission electron micrograph of Toronto/Edinburgh epidemic clone of *B. cepacia* expressing CF mucous-binding Cbi adhesin pili. High resolution was achieved by using a JEOL 100CX electron microscope and negative staining. Reprinted with permission from Richard Goldstein and Journal of Bacteriology (J Bacteriol 1995;177:1039-52).

intravenous solutions (2,26,27). The organism's unusually broad metabolic capabilities, which enable it to survive and proliferate in water-based environments, probably account for its propensity to cause nosocomial outbreaks (2,27-29). Because of its resistance to multiple antibiotics, once acquired, the organism can be difficult to treat.

*B. cepacia* is rarely found in the non-CF patient (30); however, when it is found the organism can spread to and from CF patients. Transmission between CF and non-CF patients has been associated with serious illness and death (31;Holmes et al., unpub. data) and presents a greater nosocomial threat than previously recognized. In addition, although *B. cepacia* is not a common commensal organism, hospitalized patients without CF may harbor it and pose an infection threat to vulnerable CF patients. This possible source of infection may account for the known association between hospitalization and of *B. cepacia* infection (32). *B. cepacia* may be underreported because selective media for *B. cepacia*, which greatly increase the yield (33,34), are rarely used except in specimens from CF patients.

### *B. cepacia* as an Agricultural Agent

While emerging as a human pathogen, *B. cepacia* has attracted intense interest from the agricultural industry as a possible biologic control agent. The organism, which has been shown to have remarkable potential as an agent for both biodegradation and biocontrol, is being considered as a plant-growth-promoting rhizobacterium (23,35-43).

*B. cepacia* has extraordinary metabolic versatility and can degrade chlorinated aromatic substrates (toxic compounds found in complex pesticides and herbicides, some with carcinogenic potential) for use as carbon energy sources. One important toxic compound degraded by *B. cepacia* is 2,4,5 chlorophenoxy acetic acid (2,4,5-T), a potent herbicide that is not easily biodegradable and persists for long periods in the environment (37).

*B. cepacia* can also antagonize and repress many soilborne plant pathogens. It can prevent leaf and stem blight caused by the fungus *Alternaria* by inhibiting spore germination. Economically important crop diseases such as blight due to *A. solani* and the blight caused by *A. brassicae* and *A. brassicola*, which affects the oil-producing plants rape and canola, can be controlled by *B. cepacia*. The organism is also being used to prevent the blight of ginseng plants due to *A. panax* (41) and is effective against the fungus *Aphanomyces euteiches*, which causes root rot in peas, alfalfa, and snap beans (39,40). It can prevent *Pythium* diseases of cucumber and peas, and *Rhizoctonia solani* stem rot of poinsettia (42). To prevent these plant diseases, *B. cepacia* provides a seemingly environmentally friendly alternative to potent and toxic fungicides, which cannot be broken down in the environment.

The forestry industry also sustains large economic losses from the pathogenic effects of fungi such as *Fusarium*, *Pythium*, *Rhizoctonia*, *Cylindrocarpum*, and *Botrytis*. These widespread fungal pathogens cause seedling loss in nurseries and may kill or stunt transplanted seedlings. A strain of *B. cepacia* has been developed as a successful seed and root inoculant, which can suppress these fungi on a variety of conifers (43).

Numerous patents are being sought for specific agricultural applications of different strains of *B. cepacia*. The ecologic and economic benefits could be enormous if the organism's antifungal activity is used to enhance crop yields and reduce the need for pesticides and its ability to degrade complex herbicides and pesticides is harnessed for bioremediation.

### Molecular Epidemiology of *B. cepacia*

### Evolution of Highly Transmissible Strains

*B. cepacia* isolates are closely related (a panmictic population structure) (18,31,44) and epidemic isolates are scattered throughout (18,31), as demonstrated by *rrn*-based phylogenic trees, which include large numbers of environmental and clinical isolates. Such a phylogeny indicates that in *B. cepacia* strains, genetic changes conferring high transmissibility are occurring at random, or, given the right epidemiologic circumstances, random environmental strains are innately highly transmissible. In addition, the widespread geographic distribution of different epidemic strains of *B. cepacia* would also suggest that highly transmissible strains are emerging independently and randomly.

### Taxonomy

Isolates presumptively identified as *B. cepacia* belong to at least five genomovars

(23,45,46). This group of phenotypically similar subpopulations is referred to as the *B. cepacia* complex (6,46). After multiphasic taxonomic analysis, the species names *B. multivorans* and *B. vietnamiensis* have been proposed for genomovars II and V, respectively (45). The pathogenic and epidemic potential of each of these subpopulations of the *B. cepacia* complex requires further examination. Although it appears that strains associated with *cepacia* syndrome belong to genomovar III (46), isolates belonging to each of these five genomovars have been cultured from CF patients.

### The Unusual Genome

*B. cepacia*'s capacity to propagate as an environmental microbe and as an opportunistic pathogen may be due to its possession of an unusually large (more than four times that of *H. influenzae*, two times that of *E. coli*, and half again as large as *P. aeruginosa*), complex, and variable genome. The genome contains numerous insertion sequences and is divided into one to four circular replicons (47,48). A few other bacterial species of agricultural and medical importance also have multiple chromosomes: *Brucella melitensis*, *B. abortus*, *B. suis*, *B. ovis*, *Rhodobacter sphaeroides*, and *Agrobacterium tumefaciens* (49-51).

This unusual genomic arrangement may account for *B. cepacia's* nutritional versatility and adaptability. Such a division of genomic content would allow for high levels of homologous and illegitimate recombination. The resultant chromosomal rearrangements and associated mutations could provide a basis for spontaneous “pulsed” evolutionary spurts, such as that seen from soil to the CF lung, suited for rapid adaptation to radical changes in environmental growth conditions.

### Selection of Strains for Agricultural Use

Because evolution of highly transmissible strains occurs randomly and transmission of *B. cepacia* between CF and non-CF populations can cause severe illness and death, the deliberate widespread environmental application of strains of this organism should be considered carefully. Although *B. cepacia* does not appear to survive on dry surfaces for more than 1 week, it can survive for many months in water. The agricultural application of *B. cepacia* will lead to environmental and water contamination and increased human exposure. In addition to medical, metabolic, and taxonomic issues, increasing knowledge of *B. cepacia* raises important ecological issues, including the evolution of pathogenicity and multiresistant environmental bacteria through horizontal gene transfer. The agricultural use of *B. cepacia* risks this hazard of horizontal gene transfer between the strains applied and existing soil organisms and the subsequent emergence of pathogenic, highly resistant organisms.

Recently, in an attempt to assess the human risk associated with the use of rhizophere isolates as field inoculants, two clinical isolates of different strain types from two CF patients and two agricultural isolates from the rhizopheres of rice and maize were examined (38). The clinical isolates had identical 16S ribosomal rDNA sequences, but differences were seen between the soil isolates and clinical isolates. The results were presented as evidence of evolutionary divergence of the rhizophere isolates from their clinical counterparts. Alternatively, in the light of the multiple replicon model, this difference in 16S *rrn* is most likely due to the multiple replicons carrying varying sequences of the 16s gene. Furthermore, evidence based on four isolates alone is inadequate to support the safe application of rhizophere isolates. We have seen diversity among 16s *rrn* that is unrelated to source in a large collection of *B. cepacia* isolates (Holmes, Truong, Geisselsoder, Goldstein, unpub. data).

The possibility of highly virulent strains of *B. cepacia* with the potential to survive intracellularly exists if the organism acquires virulence genes from *B. pseudomallei,* a very closely related pseudomonad. *B. pseudomallei* is a soil saprophyte that gives rise to melioidosis, a life-threatening tropical disease seen mostly in Southeast Asia. The disease has a broad clinical spectrum and can remain dormant for years before giving rise to sepsis and death. The pattern of disease produced by *B. pseudomallei* may warn of a spectrum of clinical consequences from *B. cepacia* acquisition. The transfer of genetic material between these two closely related organisms in the environment is highly probable, with the subsequent emergence of a hybrid pathogen. This speculation is supported by recent detection of insertion sequences within *B. pseudomallei* that have been identified in *B. cepacia,* including an isolate belonging to the highly transmissible transatlantic epidemic lineage (18,52).

With the potential emergence of diseases related to new and developing practices in the food and agricultural world, it seems prudent that communication and information sharing between medical microbiologists, agricultural microbiologists, and public health professionals be optimized and promoted. Meanwhile, it is impossible to identify, phenotypically or genotypically, strains of *B. cepacia* that could safely be used in agriculture. Even if environmental strains incapable of human infection could be identified, their potential to evolve into human pathogens remains. Current molecular genetic evidence indicates that the deliberate environmental distribution of the organism might pose a hazard to human health, regardless of which particular strains are selected. Until more is known about the organism and the risks from environmental application, a moratorium should be called on the widespread use of *B. cepacia* in agriculture.

This work was supported in part by a research grant award to RG from the National Institutes of Health (NIDDK RO1 DK50838).

### References

1. Burkholder W. Sour skin, a bacterial rot of onion bulbs. Phytopathology 1950;40:115-8.
2. Goldmann D, Klinger J. *Pseudomonas cepacia*: Biology, mechanisms of virulence, epidemiology. J Pediatr 1986;108:806-12.
3. Thomassen MJ, Demko C, Klinger, J, Stern, R. *Pseudomonas cepacia* colonization among patients with cystic fibrosis—a new opportunist. Am Rev Respir Dis 1985;131:791-6.
4. Sajjan U, Sun L, Goldstein R, Forstner J. Cable (Cbl) type II pili of cystic fibrosis-associated *Burkholderia (Pseudomonas) cepacia*: nucleotide sequence of the *cblA* major subunit pilin gene and novel morphology of the assembled appendage fibers. J Bacteriol 1995;177:1030-8.
5. *Pseudomonas cepacia*—more than a harmless commensal? [editorial]. Lancet 1992;329:1385-6.
6. Govan J, Deretic V. Microbial pathogenesis in cystic fibrosis: mucoid *Pseudomonas aeruginosa* and *Burkholderia cepacia*. Microbiological Reviews 1996;60:539-74.
7. Isles A, Maclusky I, Corey M, Gold R, Prober C, Fleming P, et al. *Pseudomonas cepacia* infection in cystic fibrosis: an emerging problem. J Pediatr 1984;104:206-10.
8. Tablan O, Chorba T, Schidlow D, White J, Hardy K, Gilligan P, et al. *Pseudomonas cepacia* colonization in patients with cystic fibrosis: risk factors and clinical outcome. J Pediatr 1985;107:382-7.
9. Thomassen MJ, Demko C, Doershuk C, Stern R, Klinger J. *Pseudomonas cepacia*: decrease in colonization in patients with cystic fibrosis. Am Rev Respir Dis 1986;134:669-71.
10. Tablan O, Martone W, Coershuk D, Stern R, Thomassen MJ, Klinger J, et al. Colonization of the respiratory tract with *Pseudomonas cepacia* in cystic fibrosis. Chest 1987;91:527-32.
11. Corey M, Farewell V. Determinants of mortality from cystic fibrosis in Canada. Am J Epidemiol 1996;143:1007-17.
12. Govan J, Brown P, Maddison J, Doherty C, Nelson J, Dodd M, et al. Evidence for transmission of *Pseudomonas cepacia* by social contact in cystic fibrosis. Lancet 1993;342:15-9.
13. Walters S, Smith E. *Pseudomonas cepacia* in cystic fibrosis: transmissibility and its implications. Lancet 1993;342:3-4.
14. Hearst J, Elliott K. Identifying the killer in cystic fibrosis. Understanding the genetic defects underlying CF is only half the battle. Identifying the specific bacterium infecting CF patients is just as important. Nature Medicine 1995;1(No. 7):626-7.
15. LiPuma J, Mortensen J, Dasen, S, Stull T. Ribotype analysis of *Pseudomonas cepacia* from cystic fibrosis treatment centers. J Pediatrics 1988;113:859-62.
16. Johnson W, Tyler S, Rozee K. Linkage analysis of geographic and clinical clusters in *Pseudomonas cepacia* infections by multilocus enzyme electrophoresis and ribotyping. J Clin Microbiol 1994;32:924-30.
17. Mahenthiralingam E, Simpson D, Speert D. Identification and characterisation of a novel DNA marker associated with epidemic strains recovered from patients with cystic fibrosis. J Clin Microbiol 1997;35:808-16.
18. Sun L, Jiang R-Z, Steinbach S, Holmes A, Campanelli C, Forstner J, et al. The emergence of a highly transmissible lineage of *cbl*$^{+}$ *Pseudomonas (Burkholderia) cepacia* causing CF centre epidemics in North America and Britain. Nat Med 1995;1:661-6.
19. Pitt T, Kaufmann M, Patel P, Benge L, Gaskin S, Livermore D. Type characterization and antibiotic susceptibility of *Burkholderia (pseudomonas) cepacia* isolated from patients with cystic fibrosis in the United Kingdom and Republic of Ireland. J Med Microbiol 1996;44:203-10.
20. Segonds C, Bingen, B, Couetdic G, Mathy S, Brahimy N, Narty N, et al . Genotypic Analysis of *Burkholderia cepacia* isolates from 13 French cystic fibrosis centers. J Clin Microbiol 1997;35:2055-60.
21. Steinbach S, Sun L, Jiang R, Flume P, Gilligan P, Egan T, et al. Transmissibility of P*seudomonas cepacia* infections in clinic patients and lung-transplant recipients with cystic fibrosis. N Engl J Med 1994;331:981-7.
22. Ryley H, Ojeniyi B, Hoiby N, Weeks J. Lack of evidence of cross-infection by *Burkholderia cepacia* among Danish cystic fibrosis patients. Eur J Clin Microbiol Infect Dis 1996;15:755-9.
23. Govan J, Hughes J, Vandamme P. *Burkholderia cepacia*: medical, taxonomic and ecological issues. J Med Microbiol 1996;45:395-407.
24. Taplin D, Bassett D, Mertz P. Foot lesions associated with *Pseudomonas cepacia*. Lancet 1971;568-71.
25. Jarvis W, Olson D, Tablan O, Martone J. The epidemiology of nosocomial *Pseudomonas cepacia* infections: endemic infections. Eur J Epidemiol 1987;3:233-6.

26. Anderson D, Kuhns J, Vasil M, Gerding D, Janoff E. DNA fingerprinting by pulsed field gel electrophoresis and ribotyping to distinguish *Pseudomonas cepacia* isolates from a nosocomial outbreak. J Clin Microbiol 1991;29:648-9.
27. Pegues D, Carson L, Anderson R, Norgard M, Argent T, Jarvis W, et al. Outbreak of *Pseudomonas cepacia* in oncology patients. Clin Infect Dis 1993;16:407-14.
28. Dixon R, Ksalow D, Mackel C, Fulkerson C, Mollison G. Aqueous quaternary ammonium antiseptics and disinfectants—use and misuse. JAMA 1977;236:2415-7.
29. Sobel J, Hashman N, Reinherz G, Merzbach D. Nosocomial *Pseudomonas cepacia* infection associated with chlorhexidine contamination. Am J Med 1982;73:183-6.
30. Hyde J, Humphreys H. Absence of *Burkholderia cepacia* from the respiratory tract of non-cystic fibrosis patients. Eur J Clin Microbiol Infect Dis 1997;16:253-4.
31. Holmes A, Nolan R, Finley R, Riley M, Sun L, Goldstein R. An epidemic clone of *Burkholderia* (*Pseudomonas*) *cepacia* transmitted between cystic fibrosis and non-cystic fibrosis patients. In Abstracts of the 9th Annual North American Cystic Fibrosis Conference; 1995; Dallas, Texas. Dallas (TX): Cystic Fibrosis Foundation; 1995, abstract 228.
32. Tablan O, Chorba T, Schidlow D, White J, Hardy K, Gilligan P, et al. *Pseudomonas cepacia* colonization in patients with cystic fibrosis: risk factors and clinical outcome. J Pediatr 1984;107:382-7.
33. Carson L, Tablan O, Cusick L, Jarvis W, Favero M, Bland L. Comparative evaluation of selective media for isolation of *Pseudomonas cepacia* from cystic fibrosis patients and environmental source. J Clin Microbiol 1988;26:2096-100.
34. Gilligan P, Gage P, Bradshaw L, Schidlow D, DeCicco B. Isolation medium for the recovery of *Pseudomonas cepacia* from respiratory secretions of patients with cystic fibrosis. J Clin Microbiol 1985;22:5-8.
35. McLoughlin T, Quinn J, Bettermann A, Bookland R. *Pseudomonas cepacia* suppression of sunflower wilt fungus and role of antifungal compounds in controlling disease. Appl Environ Microbiol 1992;58:1760-3.
36. Homma Y, Sato Z, Hirayama F, Kanno K, Shirahama H, Suzui T. Production of antibiotics by *Pseudomonas cepacia* as an agent for biological control of soilborne pathogens. Soil Biology and Biochemistry 1989;21:723-8.
37. Sangodkar U, Chapman P, Chakrabarty A. Cloning, physical mapping and expression of chromosomal genes specifying degradation of the herbicide 2,4,5-T by *Pseudomonas cepacia* AC1100. Gene 1988;71:267-77.
38. Tabacchioni S, Visca P, Chiarini L, Bevivino A, Di Serio C, Francelli S, et al. Molecular characterization of rhizosphere and clinical isolates of *Burkholderia cepacia*. Res Microbiol 1995;146:531-42.
39. Bowers J, Parke J. Epidemiology of *Pythium* damping-off and *Aphanomyces* root rot of peas after seed treatment with bacterial agents for biological control. Phytopathology 1993;83:1466-73.
40. King E, Parke J. Population density of the biocontrol agent *Burkholderia cepacia* AMMDR1 on four pea cultivars. Soil Biology and Biochemistry 1996;28:306-12.
41. Joy A, Parke J. Biocontrol of Alternaria leaf blight on American ginseng by *Burkholderia cepacia* AMMD. In: Bailey WG, Whitehead C, Procter JTA, Kyle JT, editors. Challenges of the 21st century. Proceedings of the International Ginseng Conference; 1994; Vancouver, British Columbia, Canada. Burnaby, B.C., Canada: Simon Fraser Univ.; 1994.
42. Cartwright D, Chilton C, Benson D. Pyrrolnitrin and phenazine production by *Pseudomonas cepacia* strain 5.5B, a biocontrol agent of *Rhizoctonia solani*. Appl Microbiol Biotechnol 1995;43:211-6.
43. Reddy M. Status on commercial development of *Burkholderia cepacia* for biological control of fungal pathogens and growth enhancement of conifer seedlings for a global market. Washington: US Forest service general technical report PNW; 1997. Report no. 389. p. 235-44.
44. Wise M, Shimkets L, McArthur J. Genetic structure of a lotic population of *Burkholderia (Pseudomonas) cepacia*. Appl Environ Microbiol 1995;61:1791-8.
45. Ursing JB, Rosello-Mora RA, Garcia-Valdes E, Lalcut J. A pragmatic approach to the nomenclature of phenotypically similar genomic groups. Int J Syst Bacteriol 1995;45:604.
46. Vandamme P, Holmes B, Vancanneyt M, Coenye T, Hoste B, Coopman R, et al. Occurrence of multiple genomovars of *Burkholderia cepacia* in cystic fibrosis patients and proposal of *Burkholderia multivorans sp nov*. Int J Syst Bacteriol 1997;47:1188-200.
47. Cheng H, Lessie T. Multiple replicons constituting the genome of *Pseudomonas cepacia* 17616. J Bacteriol 1994;176:4034-2.
48. Holmes A, Zhou H, Sun L, Goldstein R. Multiple chromosome variability amongst clinical and environmental isolates of *Burkholderia (Pseudomonas) cepacia;* Interscience Conference on Antimicrobial Agents and Chemotherapy (ICAAC) 1996; San Francisco. San Francisco: ICAAC; 1995; Abstract C51.
49. Allardet-Servent A, Michaux-Charachon S, Jumas-Bilak E, Karayan L, Ramuz M. Presence of one linear and one circular chromosome in the *Agrobacterium tumefaciens* C58 genome. J Bacteriol 1993;175:7869-74.
50. Suwanto A, Kaplan S. Physical and genetic mapping of the *Rhodobacter sphaeroides* 2.4.1 genome: genome size, fragment identification, and gene localisation. J Bacteriol 1989;171:5840-9.
51. Michaux S, Paillisson J, Charles-Nurit M, Bourg G, Allardet-Servent A, Ramuz M. Presence of two independent chromosomes in the *Brucella melitensis* 16M genome. J Bacteriol 1993;175:701-5.
52. Mark K, Titball R. The detection of insertion sequences within the human pathogen *Burkholderia pseudomallei* which have been identified previously in *Burkholderia cepacia*. FEMS Microbiol Letts. In press 1998.
